# Judicial Psychology in France

**Published:** 1859-04-01

**Authors:** 


### Art. IV.—JUDICIAL PSYCHOLOGY IN FRANCE.

We have often had occasion to admire the orderly form in which the scientific evidence in cases of disputed insanity is generally presented in France as contrasted with the somewhat disjointed fashion in which it is frequently, if not generally, elicited by a *vivâ voce* examination in our courts. All that we could wish to say on this subject is well illustrated by the report of a very interesting case published in a recent number of the *Annales Médico-Psychologiques.* The case in question is not only important in itself, but from the ample remarks which precede it, an Englishman gets without difficulty many interesting details, showing the course of practice in French courts, and the estimation in which the legal administration of the country is held by French psychopathists. We make no apology to our readers, therefore, for transferring the case almost entire to our pages. The introductory remarks being addressed to a French public, we shall replace them by some observations on the French procedure.

The case is reported by Dr. Aubanel, the principal physician of the Lunatic Asylum at Marseilles, who was commissioned to examine the mental state of the prisoner by the Assize Court at Aix, before whom the accused was brought for trial. The order for this examination was made after the trial had proceeded to the point opening out a defence of insanity, which was disputed on the part of the prosecution. It would appear from Dr. Aubanel's remarks that this course is not unusual in the French tribunals. The Doctor had two such cases in the year 1857, and he makes the practice the basis of a long argument, the purport of which is that, in his opinion, the preliminary proceedings against prisoners, in whose defence insanity is set up, are in general too hastily brought to a conclusion by what we should term the full committal of the prisoner to take his trial. The Doctor would have the defence more carefully sifted and examined in the first instance by the magistrates taking the examination, who, he thinks, are better qualified to appreciate medical evidence than juries. He thinks that if this were done, many painful cases would be stopped in the preliminary stages by a proceeding which he terms *une ordonnance de non lieu*, and the effect of which we gather to be analogous to the ignoring of a bill of indictment by our grand jury. In his remarks, the Doctor passes over entirely as an every-day occurrence of no interest the point which, to an English reader, seems really the most important—viz., that the trial of a prisoner should be suspended on the motion of the prosecution, the prisoner committed, with all the evidence against him, to the medical officer of a lunatic asylum, he being ordered to report to the Court his opinion as to the prisoner's sanity. This, at first sight, seems to be a course of proceeding perfectly inadmissible in an English Court, and opposed to some important *principles* of our criminal law. It is so familiar, however, to French minds that Dr. Aubanel evidently sees nothing in it worthy of remark. We think it must be evident to every person who has considered the subject that the guilt or innocence of a prisoner, when insanity is the point on which it turns, is very frequently decided in our Courts upon evidence of a most meagre and unsatisfactory description, as complete, no doubt, as the circumstances preparatory to the trial may have permitted; but from the unfavourable nature of these circumstances for observations of the character required, and the scanty opportunity for such observations as may be actually possible, the result is often tinged with uncertainty and conflicting opinions to a very serious extent. This state of things may lead to one of two evils—the acquittal of the guilty, or the conviction of the innocent. Of course the latter is infinitely the more serious of the two, and considering the merciful leaning of our Courts towards prisoners, there is good ground to hope that this does not often occur. But the former is a source of scandal to justice, which should be diligently avoided. The comments of some of the provincial papers upon the acquittal of James Atkinson at the last York Assizes may be referred to, as showing the feelings excited by the acquittal of a person supposed to be guilty. The whole trial, also, is a good example of a case decided upon evidence which, though strong enough to justify the verdict recorded, was yet weak enough to leave many minds in a state of uncertainty, which one feels morally sure was quite unnecessary, and would not have been left by evidence founded upon observation under more favourable circumstances. The mode in which the difficulties arising from imperfect evidence here alluded to are surmounted by the French Courts, and the way taken by them to arrive at the best evidence on the subject, does not appear capable of much improvement. As soon as a case of sufficiently doubtful insanity is made to appear to the Court, instead of allowing it to be decided upon the result of hurried observation, productive only of doubt where certainty is often attainable—instead of this, the Court suspends the trial, in order that the evidence may be completed by observations made under the most favourable circumstances. The prisoner is committed to an asylum, and there placed in the charge of a medical officer—a Government servant, if possible—but, at any rate, one who is not already committed to an opinion on the case, and this referee is charged to observe and report. His report, in the first instance made in writing, is afterwards deposed to by him in the witness-box, as evidence upon the adjourned trial, and of course he is subject to cross-examination thereon, as any other witness would be. This we conceive might readily be made the basis of a valuable and perfectly pacticable and easy improvement in the mode of conducting such trials, as that of Atkinson at York, in our courts. As a general rule, of course an application made by the prosecution in the middle of a trial for a postponement in order to get further evidence would not be listened to for a moment, and this out of regard to the liberty of the subject, because if such an application were granted, provision must be made to secure the appearance of the prisoner on the adjourned trial, which could only be done by keeping an untried man in prison an improper length of time, or holding him to bail, which is equally objectionable. But this objection does not arise in the case of a prisoner pleading not guilty on the ground of insanity, because if the plea is admitted, it does not entitle him to be set at liberty; the judgment of the court upon such a finding would be that the prisoner be kept in strict custody in such place and in such manner as to the court should seem fit until her Majesty's pleasure should be known,* therefore the adjournment of the trial of such a case is no more than a provisional admission by the court of the prisoner's plea, to which no objection can be reasonably urged on his behalf. We do not, therefore, look upon this procedure as in the slightest degree contravening any principle of our criminal law. All that seems to be wanted, is to enable the court to do in a criminal case that which they can already do in a civil case, viz., "where they deem right for the purpose of justice to order an adjournment of the trial for such time and subject to such terms and conditions as to costs and otherwise as they may think fit."† Whether this would require an Act of Parliament, we do not pretend to say, but substantially it is a matter of practice and procedure, and does not involve any change in legal doctrine or principle. No one we think will read the following report of Dr. Aubanel without feeling convinced of the great advantage which the power to order an adjournment under such circumstances must be in the administration of justice. It would appear, however, that a degree of weight and importance is in some respects attached to these medical reports in France, which in England would only be given to the verdict of a jury. Here, the report could only be considered as evidence for the jury, and that subject to the cross-examination of the person making it. In France this seems to be the main but not the only use made of the report.

Dr. Aubanel tells us that upon his first report, which was to the effect that the prisoner was insane, the adjourned trial, which should have come on at the next assizes, was further postponed until the prisoner should be cured, on the ground that the law could not try a lunatic. The English law agrees with the French in refusing to try a lunatic, but it would not admit the proposition that the prisoner was a lunatic, except on the finding of a jury. In this country we should say that the prisoner ought to have been brought before the court at the time appointed, and upon its being suggested that he was then insane, the jury should have been sworn to try the question. The doctor's evidence would have been submitted to the jury, and upon their find-

* 39 and 40 Geo. 3, c. 94, s. 1.

† Common Law Procedure Act, 1854, s. 19.

ing a verdict that the prisoner was insane, the Court would order the postponement. But in France it would seem that the Court proceeds upon the Report, treating it for this purpose as equivalent to a verdict.

Dr. Aubanel is of opinion that, in cases where there is strong evidence that the insanity of a prisoner dates from a period anterior to the crime charged against him, his trial should not be postponed indefinitely. He raises the point mainly in the interest of the families of accused persons. "It is not indifferent," he says, "to families so heavily afflicted, whether they have amongst their members a lunatic or a criminal. Insanity is no dishonour, but crime leaves an indelible stain." After stating his argument with some warmth, he says:—

"In conclusion, I would ask, Can the law be right which, in such cases as I have referred to, delays indefinitely the trial of the accused? Is it right in any case to keep hanging over the head of an unfortunate man accused of crime, but upon scientific authority pronounced insane, and as such confined in an asylum, an order which, without being a condemnation, undoubtedly casts discredit both upon him and upon his family?"

We think the law is right, and should be sorry to see it altered. A mere charge ought to cast no discredit upon a man, unless he has made default when legally called upon to meet it. It would be a most serious thing for the law to countenance any other doctrine than that which is contained in the maxim, that every one must be taken as innocent until the contrary is proved. We think that in England the general feeling of society is in harmony with the law, and that no discredit would attach to a lunatic or his family on account of a criminal charge pending the postponement of his trial. If Dr. Aubanel's argument has any weight in France, it must arise from some confusion existing in the public mind between the nature of a charge and a conviction; and it is this, if anything, which requires correction, and not the law.

With respect to the Doctor's suggestion, that effect should be more frequently given to the plea of insanity on the preliminary inquiry before trial, it points out an important difference between the English and French law on the subject. In France, it appears, a trial by jury may be avoided if the magistrate is satisfied of the prisoner's insanity. An English grand jury is not at liberty to ignore a bill on the ground of the prisoner's insanity; and hence the defence must always be made, and we think rightly so, in solemn form. With these preliminary remarks, we now proceed to give the report of the case, as sketched by Dr. Aubanel.

### *In the Assize Court of the Bouches-du-Rhône.*

CASE OF LOUIS R——.

The event under inquiry took place on the 21st June, 1857; the accused was brought before the Assizes at Aix, in the month of August in the same year, but the trial was adjourned to the next Assizes. The witnesses had been examined, the public prosecutor had stated his case, and the plea of insanity had been ably set up in defence. The trial was then interrupted by a motion of the procureur-général, who thought it his duty to claim the intervention of science, on account of the reliance by the defendant's counsel on the plea of insanity, and also because of the peculiar demeanour of the accused, who was, perhaps, suspected of simulating lunacy. The accused was admitted into the Asylum at Marseilles on the 20th August, 1857, and my examination terminated in November following, after three months' observation. This first probation was not deemed conclusive at law, and the procureur-général, in the exercise of his legitimate prerogative, wishing not to neglect any means for the elucidation of truth, resolved to have recourse to a further inquiry. It was then ordered by the President of the Assizes that the accused should be taken to the Asylum at Montpellier, there to be placed under the scrutiny of a Medical Commission, composed of Drs. Buisson and René, Professors of the Faculty of Medicine, and of Dr. Cavalier, principal physician of the establishment. He left for this destination on the 18th of December, and was kept there until nearly the middle of June, 1858. In the month of April or May the Commission made a long and carefully-considered Report, in which they adopted all my conclusions, and with me found the accused to have been insane prior to the event, while in the act of perpetration, and during his sojourn in the prisons and lunatic asylums. . . .

After this new and decisive inquiry, the accused was restored to the Asylum at Marseilles, which he entered for the second time on the 12th of June, 1858. The same day I was asked if it were possible for him, without inconvenience, to appear before the Assizes. I replied that his mental condition had undergone great improvement; that no inconvenience was to be apprehended; but that, as to his perfect cure, I could not give any decided opinion, feeling it necessary, with regard to this point, to submit him to a more prolonged observation. About a month later, the director of the asylum received a letter from the prefect, ordering his definitive committal, with instructions that, in case of his cure, he should be placed in the hands of justice. A most marked change for the better in the mental condition of the accused having taken place, I then deemed it my duty to notify this circumstance to the authorities, and, under due reservations with regard to the future, to consider him for the time as cured, and in his name to demand his appearance before the Court. He asked me, in fact, every day, if the period for this had not arrived. He was transferred from that time to the prison at Aix, and was tried before the Assizes on the 23rd of August last.

I shall not attempt a report of the trial; no new fact was produced. The demeanour of the accused was very good, and, as well as his answers, justified the declaration I had made as to the improvement in his mental condition. In my deposition I had but to give a *resumé* of my Report, and to reply, by reference to facts, to a multitude of questions put to me by the president and by the advocate. Drs. René and Cavalier came forward, like myself, to testify their complete conviction as to the real and strongly-marked insanity of the accused. Professor René stated with great force in the commencement of his evidence:—" Called frequently as a professor of legal medicine to give evidence before the Assizes, it is rare that I am not a witness for the public prosecutor; but in this case, and in the presence of facts which I have observed and studied, I place myself, without hesitation, on the side of the defence."

The accusation was maintained with considerable energy. The medical reports were neither analysed nor discussed; it was thought they might be dispensed with; this case, it was said, might be decided by the simple light of common sense, without the prejudication of scientific opinion. Reference was made by the prosecutor to Socrates, whom some had wished to accuse of insanity; to Pascal, against whom the same charge was brought; to Papavoine, whose head justly fell upon the scaffold; to Jobard, condemned notwithstanding the declarations of physicians, &c. &c. I will not analyse the speech of the public prosecutor, nor reply to a multitude of his arguments, which would not bear scientific discussion. One only regrets that in such cases medical jurists cannot at once repel the singular assertions uttered in their hearing as to the science of mental diseases—a science which some lawyers think they understand as well as those who have made them the study of their lives. One is also especially annoyed, that these unjust attacks should be made against a science which has been deemed worthy of being consulted. It must be either true or false; if it is consulted, it is surely because it is true, and deserving of confidence. Why then should it be attacked as without foundation, if its conclusions do not agree with preconceived opinions? Why go out of the way to demonstrate that its doctrines are pernicious; that there is no occasion for anticipatory medical inquiries, however solemnly conducted; that, in fact, simple common sense is all that is required in an affair of this nature ?

Two circumstances in this speech deserve special notice. The first is the construction put upon a letter of the prefect, who, seeing the improved condition of the accused, verified by the principal physician of the asylum, recalled the order for his definitive committal (*placement*) to the asylum until after it should have been decided at the Assizes whether the accused were really insane. "The jury, whatever its opinion, being incapable, according to law, of pronouncing the accused insane, it follows" said the advocate-general, in his address to the jury, "that if you acquit him he will at once be set at liberty, and will occasion fresh danger to society." This mode of viewing the question might, undoubtedly, have considerable weight with the jury! But the advocate-general could not be ignorant that in the case supposed, a most important duty would devolve upon the judicial authority—namely, that of notifying to the prefect the motives by which the jury were apparently actuated, and of causing him to feel, in regard for the public safety, the necessity for a further detention of the accused in an asylum. It is true, the man had been pronounced cured, but the cure was not confirmed; and I had reserved for further inquiry the determination to be ultimately taken as to setting him at liberty. This last question would properly fall within the jurisdiction of the administrative authority, and would be considered after the acquittal.

The other circumstance, which it is important to bring into prominent notice on account of the result of the trial, is the gist of the prosecution throughout. "The crime is *confessed,*" said the advocate-general; "it was committed with *premeditation;* the accused knew what he was about to do ; he took *the greatest precautions* to ensure success ; he *knew* that he was about to do a bad deed ; he acted thus under the influence of jealousy, and he wished to disembarrass himself of a rival ; he was never insane ; he was not so especially *at the moment he perpetrated* the murderous act. He is therefore responsible for his actions, and he merits the punishment which the law inflicts upon criminals." Will it not be tacitly allowed that the consequence of a prosecution so formulated is at least a demand for condemnation to penal servitude (*travaux forcés*) for life ? The question was, in fact, put to the jury according to the meaning of the public prosecutor, and, as will be seen, was answered in the negative as to the capital offence. A conviction was only obtained upon a subsidiary question of simple correctional police (*police correctionelle*).

The defence was a very able refutation of the arguments of the public prosecutor; it turned principally upon a judicious and eloquent analysis of various parts of the medical reports which were before the Court. But notwithstanding the ability displayed by M. Mistral, he did not achieve a complete victory; he could not avoid a *condamnation correctionelle*, which he had not the least reason to expect. The incriminated act was admitted; the public prosecutor had maintained to the last, as we have seen, the accusation of premeditated murder; and one could only anticipate one of two things—either a capital conviction, commutable perhaps to forced labour for life, in consideration of extenuating circumstances; or a simple acquittal, in consequence of irresponsibility resulting from insanity.

To the great astonishment of all who had carefully followed the trial, it did not terminate in the adoption of either of these alternatives. The president, after his summing up, put the principal question, and the only one, I repeat, which could legitimately result from the case opened by the prosecution: "Is Louis —— guilty of having attempted to murder Charles ——, such attempt, manifested by partial execution, having only failed from circumstances independent of the will of its author? You will reply *Yes*," said he to the jury, "if you think that he acted in full possession of his faculties (*libre arbitre*); you will reply *No*, if you think, on the contrary, that he was insane at the moment of perpetrating the murder." But the president did not end here; and foreseeing, doubtless, that the reply might be negative, he conceived it to be his duty to put the two following subsidiary questions, viz.: "Is the said Louis —— guilty of having wilfully assaulted and wounded Charles ——? Did he premeditate the assault and wounding indicated by the last question?" The advocate for the defence, to whom these questions had never been suggested, had no opportunity of discussing them in his pleading. This is to be regretted, for had such been the case, he would have been able to point out their full signification. It is also to be remarked, that they were not raised by the public prosecutor, neither did he hint that they might be submitted to the jury. However, the jury replied negatively as to the question of premeditated murder, but affirmatively on the question of premeditated assault and wounding. The accused was consequently sentenced to thirteen months' imprisonment! The verdict of a jury is always to be respected as such; but admitting this, may we not ask how it can happen that a man confessing the incriminated act, and that he intended to commit murder, can be guilty only of assault and wounding? If his irresponsibility (*nonculpabilité*) were admitted as to the first, it follows, as a conclusion that ought to be obvious to everybody, that the verdict should have been the same on the subsidiary questions. We may respect the verdict as the result of a conscientious conviction; but our scientific opinions being in no way modified by it, it goes to prove that the science of medical jurisprudence has still many difficulties to surmount, and that our unhappy lunatics are still ill understood by those who know not the science of observation which we profess. The rest may be judged of by perusing the Judicial Report, which is as follows :—

### MEDICO-LEGAL REPORT.

#### A.—*History of the Case.*

About two o'clock in the morning of the 21st June, 1857, frightful cries were heard in the dormitory of the small ecclesiastical college at Aix, proceeding from an alcove where a student, called Charles, slept. The superintendents and one or two of the pupils hastened to the spot. They found the young Charles seated on his bed covered with blood, and holding in his hand a sword-stick which he had just drawn from his neck. This poor young man had not seen the murderer; he had been struck while asleep, and he was awakened by a suffocating sensation as if he were being strangled. On being carefully examined, the wound appeared to commence on a level with the angle of the right jaw; passing thence through the neck to the corresponding point on the left side, it penetrated the inside of the arm on the same side, and showed itself again in the external and posterior part of that member. Its length was 32 centimetres (12½ inches). No vital organ having been injured, the wound healed without difficulty in the course of a few days.

One of the tutors proceeding towards the cries, had met Louis, another pupil, on the staircase, who asked for a key, that he might go out to satisfy the calls of nature. Suspicion immediately fell on him; but he was not to be found in the seminary, and had, in fact, made his escape by the garden. It appeared early in the morning that, after leaving, he had gone direct to the police-office, and had there declared himself the murderer, detailing at the same time all the circumstances.

#### B.—*Directions of the Order of Court.*

We, the Judge of the Imperial Court of Aix, President of the Assizes, considering—

1st. That the object of the order for inspection (*pourvoi*) is based on the necessity which exists for a scientific examination of the accused, with a view to the appreciation of his mental condition, and to determine whether he was of sane mind at the date of the crime;

2nd. That it is important in the first instance to ascertain precisely the evidence for the defence, as well for the purpose of definitively fixing its limits, as that it may serve as data for the opinion of the *expert* who may be charged with the examination of the accused.

Commission Mr. Counsellor A—— to receive the evidence in question, and any other that he may deem necessary for the elucidation of truth.

We Order, 1st—that the depositions so taken, as well as the file of proceedings to the present time, shall be placed at the disposal of Dr. Aubanel, chief physician of the Lunatic Asylum at Marseilles. 2nd—that the accused, R——, shall also be placed in charge of the said Dr. Aubanel, until the next session. We commission the latter, he being previously sworn, to submit the accused to every proof calculated to determine whether he be of sane mind, and to report the nature of any alteration that may be remarked in the state of his intellectual faculties.

We charge him especially—

I. To report, 1st—whether within two or three months preceding the crime the accused was able to take cognisance of his actions; 2nd—whether he could appreciate their tendency; 3rd—whether he had the control of his will; 4th—whether the circumstances under which the crime was committed are sufficient to account for it, leaving out the supposition of insanity.

II. To state all the indications proper to guide the Court in coming to a conclusion on the question, whether at the moment of perpetrating the crime, Louis was, or was not, in a sane state of mind, and what is now his actual condition.

III. To report his observations and opinion on the above questions, in order that such report being testified may be ultimately proceeded upon according to law.

C.—*Examination of the facts appearing upon the proceedings.*

In order the better to appreciate these facts, they should be divided under four heads:—The first comprising those relating to the infancy and youth of the accused up to the time when he put on the robe of a priest; the second, those peculiar to the period of the last months preceding the event; the third treating of those which characterized the perpetration of the murder; and, lastly, the fourth, including those noted in the prison at Aix, between the event and his appearance before the Assizes.

1. *Antecedents of the Accused.*—Louis belonged to an honourable family, which had experienced great misfortunes. His mother, long a widow, had suffered great poverty, and could not have brought up her children without the assistance of various charitable persons and establishments. His maternal grandfather was quite imbecile (*tout à fait fou*); according to Dr. d'Astros, he perpetually exhibited a gaiety of demeanour resembling that of drunkenness. One of his maternal uncles presented the same symptoms. One of his cousins threw her self into a well; and, although apparently a very sensible woman, she is subject to fits of melancholy, which dispose her to suicide.

When he was about three years old, the accused, according to the statements of his family, would appear to have been afflicted with a very serious illness of a cerebral nature, leaving him slightly deformed. At ten years of age he left his father's house, paid a visit to the Curé of Cabriés, went thence to Marseilles, and returned to Aix, without any discoverable motive for so doing. He declares now that he stole a twenty-franc piece from the Curé of Cabriés. He also accuses himself of a theft of about 100 francs, committed, when he was about ten years' old, at the house of a tradesman at Aix, where he was employed. He states that he laid out this money partly in the purchase of sweetmeats; but none of his family knew of these thefts at the time, and the persons stated to have been robbed look upon the thing as impossible.

His mother, wishing to devote him to the Church, deemed herself very fortunate in obtaining his gratuitous admission to the small seminary at Aix. He entered there, in the first instance, as a day-scholar, and afterwards as a boarder. He has been educated there from about his twelfth year; this year he took his place in the rhetoric class. Some persons who knew him, both before and after his admission to the seminary, always considered him of an odd, light-headed character. He was in the habit of making grimaces at passers-by, and mocking them. His conversation was inconsistent and childish. He would often yell and cry out frightfully during the night, and at such times his relatives would come and calm him, telling him not to be afraid. He has been known, something about two years ago, to shut himself up in his room, and there utter oaths, and imitate all the street cries. At other times he abandoned himself to motiveless transports, overturning articles of furniture in his way. One of the witnesses declared that his ideas were never consecutive, and that he always considered his organization imperfect.

His conduct in the seminary is stated to have been always excellent: he has never given occasion for the least reproach on the ground of immorality or irreligion; he was known to be sad, pensive, and melancholy, of great feebleness of character, exhibiting exaggerated scruples, appearing sometimes odd, often inconstant in his determinations; but he was very pious, very docile, and very submissive to authority; he was capable of giving good advice; he pronounced at need wise and enlightened counsel. There, as in the world, he was remarked for his infantine manners. Of excessive and even feminine sensibility, he was very grateful for kindness, showing it very warmly, and being at all times very loving and very caressing. He exhibited a strong desire to be loved and humoured. He had a heated and wandering imagination. Ideas and projects of the most opposite nature sometimes followed one another in his mind. His capacity for study was ordinary and satisfactory.

In consequence of the feebleness of his character, of his unreasonable scruples, and of his continual recourse to his superiors for advice as to many of his actions, it was only with much hesitation that he was invested with a priest's robes: he was postponed from one period to another; this troubled him, and rendered him very unhappy, judging from a letter written to him by one of his relations, who was a priest, in order to reassure him and encourage him to be patient. However, his piety and his general conduct leaving nothing to be desired, he was authorised to take the gown at Christmas, 1856.

Up to that period, no one in the seminary had remarked in him any certain indication of mental alienation. One of the witnesses states that his mind was not impaired, but that he always made himself remarkable in his recreations by the originality of his actions. He had a dreamy and preoccupied air. He sometimes quitted his friends rudely in the middle of a conversation. Another witness states that he was not insane, but that he committed acts of eccentricity.

2. *Facts observed after his investiture.*—Dr. d'Astros mentions having had him under his care for about six months, for a nervous convulsive disease, similar to epilepsy. Three months afterwards he had him under his care for a serious attack of erysipelas in the face and scalp. This was followed by a violent delirium, which gave way under the influence of profuse nasal hæmorrhage. He was cured of this illness in about twenty days; but since that period he has continued to suffer in the head, and to complain frequently of pain in the frontal region.

After this illness, it was observed that his ordinary scruples increased, and his sadness became greater. A great change took place in his conduct: he became neglectful in the performance of his religious duties; he was more distracted and taciturn; he conversed frequently during the time for study, and he appeared absorbed in some great preoccupation. He went frequently to lay open his griefs and troubles to the Superior. He confessed according to custom, but he did not communicate so often as before. It was generally thought to be in consequence of excessive scruples that he did not communicate oftener.

On the 25th May, when he had to make a philosophical composition upon Jeouffroy, it was seen with great astonishment that his theme contained sentences full of exultation against the mysteries of religion. He was expostulated with on the subject, without, however, attaching any great importance to the singular ideas he had expressed. But, two days later, on the 27th, a letter of his was found in the dormitory, which strangely surprised his Superiors. This letter read as though written to an accomplice without, who was favourable to his designs. It is as follows:

"You guess why I write to you. You can send me by the bearer of this letter the weapon I have asked for. Take care that it is well sharpened, for it is important that I should succeed. I must not fail. I foresee the place where I shall meet the papistical wretch alone; and, I assure you, he shall have it in real earnest. These monsters! they made me swallow whatever they thought fit; but I know it now, I hope, and I will only believe what I like! I reckon, then, on your compliance. You know the day, the hour, and the place, when and where to expect me after the happy blow is struck. Take care to have the clothes and everything necessary for my flight.

"Ah! I assure you, on my soul, I will give it him in earnest—the papist rascal—the tyrant! and if the other papists do anything to stop me, I swear that they shall fall dead at my feet. I will say no more.—You know who is speaking to you.

"P.S.—I forgot to tell you to warn the good pastor, N——, that I will abjure wherever he wishes all the idle stories with which my mind has been filled."

On the address of the letter were the words—"You know where to take it . . . and the sooner the better."

The day after this letter fell into the hands of the Superiors, Louis disappeared, without its being known during the whole day what had become of him. He was sought for diligently, even in his mother's house; but in the evening he was found squatted under a staircase, in a kind of wood-house, where he had passed twelve or fourteen hours without anything to eat. He only left this hole after long and pressing exhortations. It was considered on this occasion whether this unreasonable conduct was the result of a kind of shame and confusion on account of his having written the letter referred to, or whether it was simply from feebleness of mind. Whatever was the cause, however, it was from that time settled that he could no longer be a priest. But, inasmuch as he was pious and without means, and as he appeared to have a taste for a monastic life, he was advised to enter the community of the Brothers of St. Jean-de-Dieu, which he seemed heartily willing to when the vacations occurred at the end of the year.

It is important now to establish one fact, namely, the friendship which Louis had for some time conceived towards young Charles, he who had almost perished under his attack. Within a short period he had made various inquiries about this young man, and appeared much interested in him, asking if he stood well in his class, and saying freely amongst his comrades, that he was a fine boy, that he bore a good character, that he was very agreeable, and that it would give him great satisfaction to form a mutual attachment with him. He sought opportunities of being with him, of talking to him, and of telling him that he wished to become an intimate friend. He deprived himself sometimes of his luncheon in order that he might give it him. Charles looked upon all this as a weakness, and joked about it with his comrades. He did not attach any importance to it, and did not absolutely repulse the testimonies of affection of which he was the object. Sometimes at bedtime Louis entered the alcove where this young man slept, and talked a short time with him; once he tickled him in order to awake him, occasionally on leaving him he kissed him on the cheeks and even on the mouth. Latterly he wrote to him, almost daily, letters filled with exalted and affectionate sentiments, and expressions of ridiculous and exaggerated love. One day he pricked his finger in order to write some words with the blood which came from the wound; another day he sent him an image of St. John fishing for hearts with a line. He had inscribed on the image this legend: "O that I could thus take thee!"

Charles replied to none of these letters, which much vexed him. On his part Charles had no sympathy for Louis; his character did not please Charles in the least. However, he one day gave him a note which we shall speak of presently on account of the important bearing it seems to have had upon the ultimate intentions of the accused: but, before passing to a new class of facts, we take it as proved by the various depositions referred to that the accused was never considered by his comrades as guilty of any act of immorality. Charles does not deny the inexplicable attachment of which he was the object; but neither does he state that the accused ever made him any dishonest proposal, never did any lewd or immoral word issue out of the mouth of the accused, nor did he ever manifest towards Charles any gestures or actions denoting bad intentions. His affection was well known in the seminary; the Superior even was not ignorant of it; but although he expostulated as to its ridiculous extent, neither he nor any one else saw anything wrong in it; he was convinced that it covered nothing disgraceful, and that it was simply the result of a sensitive and affectionate character.

3. *Facts relative to the perpetration of the murder.*—On the morning of Saturday, the 20th June, the accused having obtained permission to go out, went to his mother's house, in order to get a sword-stick which he knew to be in one of the garrets. He got it, and returned to the seminary, carrying it under his gown without any one seeing it in his possession. He then hid it in his mattress. About two o'clock in the afternoon, he received a note from Charles, which the latter had sent through a mutual friend, saying, as he gave it, that it was a reply which would make Louis *bisquer*. This allegorical note enclosed two hearts intertwined, under which were the words, "One for evermore." Beside this was a third heart pierced by a sword, under which were the words, "This would be too much!" Towards the evening of the same day, Louis confided under promise of secrecy to one of his comrades a letter remarkable alike for the superscription on its envelope, and for its contents.

1st. *The Superscription.*—"If at noon you see me with the community doing as others, you are to return me this document. But if it should happen otherwise, you are to break the seal, and read it yourself, and to such other persons as you may think proper. But if I am here at noon, you are to return it to me, and I rely on your word."

2nd. *The Contents.*—"Do not believe me so guilty as people say. . . . No one knows my reasons for acting thus. . . . Ah! if he had fulfilled my hopes, how happy we should have been. . . . But I always thought so . . . this would have been too great a happiness for thee. Louis! . . . that which will console me in my fetters, will be the thought that no one will enjoy any more than myself that which I have loved with an unrequited passion. . . . But what matters it, I love thee, and nothing shall efface thy memory from my heart, thou, the victim of my love . . . ."

It was, as already stated, about two o'clock in the morning of the 21st June, a few hours after having written this letter, that Louis committed the murderous attempt before mentioned. After the perpetration of the murder, he fled from the seminary. On arriving, without either stockings or cravat, at the residence of the Commissary of Police, he made himself known, and how he had committed the murder.

"Two months ago," he said, "a homicidal idea took possession of my mind. I endeavoured several times to put it in execution, but various circumstances prevented me. It was upon one of my professors that I wished my blows to fall. Yesterday, for the first time, I conceived the project of immolating one of my comrades—he for whom I had the greatest affection in the world. I neither can nor will I tell you the motive which determined me. I took my precautions yesterday.

I had provided myself with a weapon in the morning, which I intended to have used upon one of my professors. I kept myself awake with difficulty during the night, and I struck my victim at two o'clock, while in a deep sleep. I lay down again; but the piercing cries of the wounded youth becoming insupportable, I rose, and came out feigning a call of nature, after having told the superintendent that I did not know whence the cries proceeded."

Several persons in the house had remarked a change in his behaviour following upon his illness, and had observed that his peculiarities had latterly increased. One of the superintendents declares *that he was no longer the same as usual,* without being able to appreciate the cause. Excepting these phenomena, no one in the establishment had considered him insane before the perpetration of the murder. His peculiarities were regarded as the result of a whimsical and ridiculous character. However, the Superior now considers this murder as "an incident" in the life of this young man. "It is," says he, "the result of a species of *homicidal monomania,* arising in a distaste of life produced by his sufferings, the misfortunes of his family, and the uncertainty as to his own future."

4. *Facts subsequent to the murder.*—On the 22nd June, during the first examination which he underwent before the *juge d'instruction,* he admitted, as he had already done before the Commissary of Police, all the circumstances of the murder.

"I sought," said he, "almost in these terms, to form an intimate friendship with Charles, because his appearance pleased me, and his character appeared to accord with mine. I endeavoured to make him understand my sentiments towards him by means of my conversation, gestures, general conduct, and sundry notes. I have written to him ten or a dozen times, perhaps. My letters contained various matter: I expressed my sentiments of friendship for him, and my desire to form an intimate acquaintance with him; but they contained no immoral thought—no bad expression. I had long had a project of killing one of my professors, but that of killing Charles only entered my mind in the afternoon of the 20th June. I cannot tell you the motive which determined me; that is my secret; but it was not that which you suppose. I confessed three days before, but I have not communicated since the Ascension. It is more especially since that period that the homicidal ideas have beset me. I understand quite well the responsibility which hangs over me. I do not believe I was insane (*fou*). I acted with premeditation, and I prepared myself to commit murder, although the weapon with which I provided myself was not intended in the first instance to be used against him whom I have wounded. My professors were not always quite just towards me. It was not in consequence of a distaste for life that I gave way to the act which I now regret; faith had not left me; I knew quite well that these homicidal projects were mortal sins, but, nevertheless, they continually came into my mind."

On the 25th June, during his second examination, he said that the letter found in the dormitory was his, and that he had thrown it down in the hope that it would cause his expulsion. The pretended interview with a Protestant minister was pure invention, as also the complicity which was implied with some one outside. He had not lost the wish to become a priest, but he considered himself unworthy of the sacred office—not on account of immoral conduct, but because of many weaknesses before which he had fallen, and of numerous infractions of the rules of his order, which made him think that he was too unsteady for the ecclesiastical state.

On the 26th June, during his third examination, he acknowledged the advances which he had continually made in order to gain Charles's affections, the visits which he made to his alcove in the evenings, and the caresses of which mention has been made; but he denied any immoral act or gesture, as also all lewd thoughts.

"I felt," said he, "an attachment for this young man which made me seek after his friendship without knowing why; it was a sentimental feeling, and nothing more. I did not desire or intend to express more than this in the letter which I wrote the evening before the event. I admit that the expressions there made use of may appear impassioned; but there are good passions as well as bad, and that which I felt was not bad, I assure you. I did not wish to kill him for fear that he might reveal our relations (*liaison*), considering that I had proposed nothing bad to him. Neither had I any thought of killing him in order to prevent him ruining himself; it was not in consequence of jealousy, caused by the receipt of his note—that was but an accidental cause, and not the true cause; as to the latter, I only know it, and I will not disclose it."

Lastly, on the 1st July, during his final examination, he declared that he had committed the two thefts when about ten years of age, which have been already referred to. He again repelled the imputation of any immoral ideas in connexion with his love for Charles: his caresses, he repeated, had nothing lewd in them; they were simply the testimony of a pure affection.

"I admit my crime," said he, "but I deny all the depraved ideas now imputed to me. The motive which induced me is my secret. As to my love, I also admit it; I loved the young man—I do still, and ever shall love him."

Dr. d'Astros, physician to the prison, having had occasion to see him professionally while he was in custody, observed that his conduct was very extraordinary. Sometimes he would walk off in the middle of a conversation, notwithstanding all attempts to retain him. He told the Doctor that something quite indefinable was passing in his head; that he did not feel quite master of himself; and that, as he was left too much at liberty, he could not answer for what might happen. Dr. d'Astros considered him as affected with both suicidal and homicidal melancholy. He appeared to be tired of life, and said he should be very happy if some one would cut his head off.

The gaoler observed him to be sad and morose on the day of his imprisonment. In a short time he exhibited various eccentricities, such as saying that he was followed by a spectre; another day throwing dishes against the wall, breaking a water-bottle and other articles, and cutting shrubs in the garden. He called himself sometimes a cardinal, and superior to the archbishop. In speaking of his own case, he said, "They will kill me because I have killed my equal;" citing, in support of this, a passage of Scripture. His conversation in prison was sometimes very consistent and sensible; but at other times, when spoken to, he was incoherent, heaved deep sighs, or was silent. One day he walked about rapidly in the corridor, and only stopped when exhausted by fatigue and perspiration.

### D.—*Direct Examination of the Accused.*

Louis is eighteen and a half years of age, rather thin than stout, and of average height. His air is that of a bashful man, and one unaccustomed to society. His physiognomy is timid and generally mild, but sometimes rather harsh (*dur*) when he frowns and appears to be preoccupied. The whole of his left side, but especially his mouth, is decidedly deformed. His eyes are deeply set in their orbits, his forehead low and undeveloped. At times slight chorea-like muscular contractions of his face may be observed. His skin is of a good colour; his temperament nervous-sanguine, with great nervous predominance. His sensitiveness (*sensibilité*) appears to be excessive: the slightest reproach torments, irritates, and exasperates him. He appears to be naturally disposed to exaggerate all his impressions. He is of a distrustful temper, and at present but little affected by tokens of affection offered to him. Believing himself to be deceived by all the world, he distrusts even the affectionate letters written to him by his relatives.

Within a few days after his admission into the asylum, he tore in pieces a blouse which he brought from the prison. About a month later, he also tore up a cravat and the facings of a vest belonging to the establishment. He had completely torn up this garment before the arrival of an attendant who would have prevented his continuing to do so. He was calm and without excitement at the time. On being interrogated as to this act, he replied, that he did it without any motive or object, and without being able to resist doing so, although he knew it was wrong.

He was seen several times to walk about with incredible speed and with a sort of exasperation, making gestures, raising his hands to heaven, muttering and appearing a prey to some irritation; but he generally appeared thoughtful, sad, and preoccupied. He had little communication with the other lunatics; he lived apart, being unwilling to mix with *madmen* or to talk with them. A peculiarity remarked in the seminary—namely, that his conversation was not always consistent; that he would abruptly leave one subject for another, and would walk away in the midst of a conversation—was several times verified in the asylum. He was alternately happy and miserable; at times laughing immoderately, or smiling in the midst of a serious conversation; at other times he appeared exceedingly dejected, and a prey to a profound melancholy.

The same day that he was admitted, he conjectured his position, and understood that he was with madmen, although in his department there were but a few patients, and they very quiet:—

"Why," said he to me, some days after, "am I brought to a madhouse—to a place where everybody but yourself is insane, and speaks without reason? My reasoning, *clear* and *good*, should satisfy you that I am not mad. I have strange ideas and severe pains in my head, but I am not so mad as those who have sent me here. It is repugnant to me to wear linen which madmen have soiled with the perspiration of insanity and disease. I am placed here probably in order that I may suffer a few months longer; or, perhaps, by a refinement of cruelty, I am allowed to breathe the pure air in order to render me the more miserable by afterwards thrusting me into some horrible dungeon."

Another time he said to me—

"How can I trust any one, since even my advocate has betrayed me, having dared to say that I was mad, and that there were several kinds of insanity? It is true I was for some time *over-excited*, *raised beyond myself;* but lost my reason, oh no! never!"

He complained, from the first interview I had with him, of suffering in his head. "These headaches," said he, "commenced at the time I had the erysipelas; they give me horrible pains at intervals." He almost always feels a kind of uneasy heaviness. He adds that he has invariably an indefinable sensation about his forehead. Very often these pains in the head rendered him incapable of working, reading, and doing his duties in class. He was obliged sometimes, at the seminary, to go and lie down in order to obtain relief. Under the influence of these sufferings, the most strange and extraordinary ideas came into his head, ideas which he never had before his erysipelas, that serious illness in his head which kept him in bed nearly a month. These ideas, on the other hand, seemed to aggravate his terrible headaches, which he now endured almost continually. Sometimes he experienced, in addition, a sensation of great fatigue and general lassitude, general uneasiness and want of sleep. He told me that, in order to allay these sufferings, he needed to be allowed liberty to walk in the fields, and to breathe the pure air. He asked me earnestly to let him take some walks in the garden, thinking that the mere sight of trees and verdure would do him good; he even asked me to tie him up to one of the trees in the garden if I was afraid of his escaping. After about ten days' observation, I allowed him daily walks, and I ordered him warm baths. He was very grateful for this favour, thanking me profusely; and he assured me that he experienced both physical and moral relief from this treatment. Purgative pills were also administered occasionally, in order to overcome obstinate constipation.

Besides these physical sufferings, and those instances in which he tore his clothes, the intervals of exasperation which have been noticed, a few peculiarities of manner, and alternations of mirth and sadness, I have observed no other organic perturbation, nor any external manifestation of moral or intellectual disorder. All his functions worked well; his appetite was good and his pulse normal. His nervous system, however, was over-excited; he seemed to experience what are commonly called nervous contractions, twitchings, and restlessness. The muscular motions of the face seemed to partake of the same nature. This state, together with his habitual air of preoccupation, gave him occasionally a strangeness of physiognomy which can neither be described nor explained, but which struck me as proclaiming an abnormal and peculiar moral condition. In other respects he was calm and submissive. He conversed generally without incoherence of idea, without agitation, and without any apparent disturbance of his faculties. His memory was good, his recollections perfect. reason, however, did not appear well developed, his judgment leaving much to be desired. His actions of common life were not disordered, but were generally those of perfect sanity. In a word, there did not exist in the accused that aggregation of symptoms visible to every eye, which alone constitutes madness in vulgar estimation. Nevertheless, persons in the habit of seeing him, without calling him insane, could not fail to see that there was something about him that was unusual and peculiar.

Interrogating him minutely in various interviews on his previous life, his most intimate thoughts, his habitual occupation, and on the singular ideas which, according to him, most frequently took possession of his mind, the following details were obtained in almost as many words.

"When very young, he had to undergo great trials; his family experienced one after another great sufferings and afflictions. His mind was early affected by them, and he became in consequence more thoughtful than other children, and averse to childish amusements. He was weary and unhappy when his playfellows were happy and contented; his mind was always depressed and sad. This was more especially the case during one or two years, when his family having lost the last remnant of their fortune, came to actual want and misery, and had to undergo in consequence great privations. At Christmas, last year, he was made happy by being authorized to take the gown, which gave him lively satisfaction. Notwithstanding his happiness, however, he was not without scruples; he asked himself, sometimes, if he was worthy of the robe with which he had been invested, and whether in that career the assistance of God would ever abandon him. His doubts in this matter were not to be wondered at, as he was always by nature very right in his dealings towards God.

"After his attack of erysipelas, strange ideas came into his head; he could only think of the most extraordinary things, dreaming now of immense riches, now of the supernatural power of becoming visible and invisible at will, now of changing his religion and now desiring to make himself celebrated by some great *éclat.* One day he took it into his head to sell himself to Satan; and in order that the devil might find nothing to prevent his fetching him, he laid aside everything about him that had been blessed by the Church. Another day, the idea of abjuration presented itself; he thought of becoming a Protestant, feeling no more for the time the same faith in his religion that until then had always animated him. It was under the influence of this idea that he wrote the letter found in the dormitory, and after which he hid himself in the wood closet under the staircase. Another motive partly induced this act: he wanted to do something *extraordinary;* it would be very *extraordinary*, he thought, for a young student to write and meditate such a wonderful thing. He did not know very well why he had hidden himself in such a hole, his head was in a state of *confusion and stupidity* that day, in a state too extraordinary to be described, he having remained all day lying upon very irregular pieces of wood without having felt the least physical want or desire to move.

"He always took pleasure in singularities; but latterly his ordinary taste for such things became exaggerated, he was pursued more and more by extravagant and unusual ideas. The extraordinary pleased him above everything; thus he was continually tempted to speak against religion, to speak ill of that which was going on well in the house, and to change his religion and become Protestant: he was also often beset with a desire to kill some one, and could not get rid of it. At other times he felt an inclination to commit suicide, to swallow poison or to allow himself to die of hunger. At intervals his predominating idea was to leave the world and to retire to the Chartreuse, or to embrace one order or another. Sometimes he was on the point of running away, to roam about the fields without knowing where he should go. He declares that on the very day of the event, wearied by the attacks of these ideas and being tired of life, he swallowed some chemical substance which had been given to him by a comrade in the hope of poisoning himself; he took it in the morning and afternoon without succeeding to do himself any harm. In fine, thousands and millions of extravagant ideas successively possessed his mind—sometimes even that of putting on a cardinal's robe, or of clothing himself as a woman and walking about so disguised.

"Another notion, equally as extraordinary as the preceding, took possession of him about the same time—namely, that of his passionate attachment to Charles, one of his comrades. The manners and personal appearance of the latter pleased him and inspired him with sympathy. He felt towards this young man an irresistible transport, lying in wait for opportunities of seeing him and speaking to him, seeking to attract his good opinion, and showing on every occasion the desire to attach himself to him. He continues to love him, notwithstanding all that has happened; one thing that troubles him at the present time is the thought that he is detested, hated, and execrated by him whom he has so passionately loved. What is the reason of this love? He has been asked the question a million times, he was asked it at every turn, and he asks himself, but he cannot give any account of it or explain it. It was something that carried him away, something that constrained him involuntarily. It was a *very ardent and violent affection*, such an affection as no one ever before experienced. This affection mastered him as absolutely as did, at other times, the crowd of singular ideas which came so often into his mind.

"It has been supposed that this affection had in view an object at once frightful, infamous, and horrible: this is a mistake; such a thought never entered his mind, he could not have it, being ignorant that man was ever capable of such horrors, and having only learnt in prison the nature of the infamous act which was supposed to be the motive of his passion. He repelled with indignation accusations of this nature. 'My judges,' said he, 'seem to have regarded it as evident, as demonstrated; it is false, completely false.' In his interviews with Charles, he had never made use of an equivocal word; never had he suggested anything to be ashamed of, nor did he ever attempt anything like an indecent gesture. He wrote to him with passion, he sought his society, and he sometimes embraced him, but without any other object than that of a pure friendship, or, in other words, than treating him as an intimate friend and loving him as a brother. He asserts solemnly that, in truth, no lewd thought ever crossed his mind in connexion with his young comrade.

"These thousand extravagant ideas which entered his mind threw him into a frightful and horrible state. Sometimes they were so powerful and distressing, that he could not work, so great was the confusion into which they plunged his faculties. He had hideous dreams during the night: he awoke frequently, thinking he saw the devil, whom he had oftentimes invoked; believing he heard a noise approaching his bed; imagining that some one came to spy him during his sleep; hearing people speak, and even seeing people fly at the moment of his awaking. All this agitated him, and made him very unhappy. It also troubled him very much to perceive that his piety grew weaker day by day: he experienced distractions in the exercises of religion which he had not known before; he was not even collected at the most solemn moment of the Mass; his faith was no longer profound; the most serious doubts assailed him, notwithstanding his unceasing efforts to drive them away. During the celebration of the Mass, such ideas would cause him to tremble, and he sometimes wished that the earth would open and swallow him up. He finished by no longer approaching the sacred table, partly on account of his absorbing preoccupations, partly because of the change which his belief underwent at intervals, and lastly, because he believed himself unworthy, considering the homicidal ideas and other singular thoughts which continually occurred to him as incompatible with the holy sacrament. He repelled these ideas incessantly and unrelaxingly; but they reappeared without cessation, and, in so doing, plunged him in unheard-of suffering—sufferings beyond endurance.

"For some time after these ideas came to him, he found relief in confiding all his thoughts to his confessor, and obtaining from him the consolation that he needed. He returned to his duty more calm, less troubled, and better able to drive away the singular ideas which made him so unhappy. He also frequently consulted the Director of the Seminary, by whom he was very well received; he was equally confiding towards him, and received good advice concerning both the exaggerated scruples which tormented him, and his indefinable moral condition. This condition caused him at intervals such inquietude, that he does not know what he would have done to rid himself of the ideas which beset him. He prayed God to cause his torments to cease; he commended himself to the blessed Virgin; he invoked the intercession of divers saints; he performed several novenas; in a word, he had recourse to all the prescribed forms of religious supplication; but it was useless, he obtained but momentary relief; the same ideas returned with the same force, notwithstanding his prayers and the sage counsel which he received from his Superiors. His faith was more and more shaken; he asked himself sometimes in his affliction, why God thus abandoned him, and put him to such severe trials.

"After the scene caused by his letter against Papists, he was very much grieved to see that it would be impossible for him to follow the vocation which he believed he had for the sacred ministry. But the paternal advice which was given him on this occasion appeared to recall him to a better state. He understood it, thanked his Superiors for it; he was completely resigned to what was required of him, and he was satisfied with the proposition that he should enter the community of the Brothers of St. Jean-de-Dieu. This calm was unfortunately of short duration; he soon fell again into the same torments and perplexities; the strangest ideas again assailed him, but the idea of homicide predominated more than ever, but without his knowing against whom he should seek to put it in execution. He thought for some time of killing one of the professors at the seminary.

He had come to no determination when he went out on the morning of the 20th June to get the weapon which he hid in his bed; he went out under the influence of general ideas of homicide, but without having determined to put them in force that day, and, above all, against his best friend. It was in the afternoon of this day that he received the allegorical note from Charles which he regarded as a sign of contempt, and feeling more unhappy than usual, and wounded in his affections, he conceived on a sudden the idea of killing him with the weapon he had at his command. From that time he had but this one thought; he was possessed by it until the evening; he wrote the passionate letter, acknowledging himself the author of the projected murder, and, without fear of the consequences which he was about to draw down upon himself, he thought only of his project until the fatal moment of its perpetration.

"The hurt which his feelings had received, doubtless, had something to do with his ultimate and dire determination; but this motive was only secondary; it would not have induced him to commit such a lamentable act without the dark preoccupations to which his mind had been subject, without those habitual ideas of homicide which made him so unhappy, and of which he could not rid himself. In truth, no ordinary motive would have sufficed to lead him to such a melancholy act; he but obeyed, in fine, the wish to make himself remarkable —that desire for notoriety which left him no repose. He wished to kill his best friend, because such a murder would be more astounding than any other; but in all probability he would one day have killed one of the professors, but for the note which fixed his ideas on Charles.

"The secret motive of which he spoke when examined, and which he refused to disclose, had in reality no existence; he wished then, in speaking thus, to aggravate his situation, by inducing the belief of a real motive for the crime of which he was guilty; but he was offended to find it supposed on account of this reticence that he was actuated by so infamous a motive as that of which he was accused. There was nothing in his love which he was ashamed to confess; he had never concealed it, and he had no fear that his friend would accuse him of things which never entered his mind.

"This murder, which appeared so monstrous, was not an act of wickedness on his part. It was contrary to his whole character; and that he should have been led to commit such a deed, was not only *most extraordinary*, it was *incomprehensible*. To hate evil, and to be *obliged* to do it, is a horrible thing! Such a thing could only happen by the direct permission of God. Formerly he had been happy in the seminary; he had good desires, his faith was fervent, his prayers were granted, and his felicity was at its height when, having been invested with the gown, he saw a bright prospect opening before him. Those about him in the seminary loved him, consoled him in his troubles, and gave him continual proofs of friendly interest. Why was this changed? Why was his soul abandoned to the thousand singular ideas which had tormented and did still torment him? It was a long time before he was able to explain it, but he now saw clearly that

God had given him up, that he wished to punish him, although he knew of nothing in himself that appeared to merit such chastisement. He no longer prayed, because God would not listen to him; he thought it useless to attend Mass, he had nothing to hope for in so doing; he wished to forget God, as he had been forgotten of him. Man had equally abandoned him; those whom he loved, hated him; the letters he received no longer expressed the truth, and the pledges of affection offered to him were false; even his relations did not love him. He would not receive visitors, because, in the first place, he had no real friends; and secondly, because they only gave him pain, by recalling a state of happiness which was his no longer: he had no further hope —heaven, earth, and hell had conspired against him.

"Since everybody believed him capable of meditating the infamy of which he was accused with regard to Charles, and since they persisted in this accusation, notwithstanding his denials, his protestations, and the absence of all proof, let them make haste to punish him and cause him to endure the most atrocious suffering; he would support them with courage, although he had merited death beneath the stroke of such an execration. He was guilty towards Charles, not for having loved him, but for having attempted his life. He could understand that for this he merited punishment. One of his dreams now was that he might die under the hand of him whom he had loved and did still love with so much ardour. 'Yes, Charles, I love thee! yes, I love thee,' said he in a letter, 'but what matters it that thou lovest me not? There was a power within me which compelled me to do it, and I could not resist it. Even now I love thee, although thou hatest me, abhorrest me, and hast not forgiven me. Yes, I love thee in spite of myself. My ambition is that thou shouldst come and pierce my heart, and that I should die with the words "Charles is avenged." But this would be a happiness too great for me; I cannot even hope for an end so sweet.'

"When he thought of his sufferings, and considered that God had not listened to him, and that it was by his permission that the homicidal ideas came into his mind, his feelings were something extraordinary—he experienced extreme excitement *and mental convulsions unspeakable.* All his nerves, all his body, said he, were put in motion. It was an excess of cruelty on the part of God, that he permitted persons to write to him, and to ask to see him. He wished to fill up the measure of his grief by permitting to be recalled to his memory pleasures which were lost to him for ever. God had placed him in such a state that, when any extravagance passed through his head, he must do it without the power of resistance. Was it not cruelty to have put him in such a state? He could not tell what his head was made of, it was always working. Occasionally he was tranquil, but at intervals he neither knew what he did, what he should do, or of what he thought. When he made gestures and contortions with his arms, it was because he was agitated by internal convulsions which led him to make such movements. Many a time he had an impulse to do wrong! He would do it if he could. Oh! how frightful was such a state! he would get rid of his reason if he could ever do so."

Such is the statement of the accused, and such in substance are his thoughts, as I have been able to gather them, in the numerous interviews I have had with him. Having frequently led him over the same ground, I have always obtained the same answers. He has never varied his story, and he has always spoken to me with confidence, and appeared perfectly sincere and convinced of that which he related. The various letters which he wrote in the asylum expressed the same thoughts. It was observed that he rarely said his prayers before retiring to rest; he never asked to go to Mass; he went ultimately with the others, after I had expressed to him my astonishment at his conduct, but I was satisfied that he paid no attention. He looked about distractedly from one side to another, without following the service and assisting therein, without the least sign of feeling. He repeatedly said that God having been the cause of his misfortunes, and having completely abandoned him it was useless for him thenceforth to pray. He obstinately refused to speak to several persons who called to see him, saying that they only came to mock him and make him more unhappy. Everybody, even his own family, had devoted him to execration, and, therefore, why should they come to see him? His sisters wrote him letters full of affectionate consolation; but their kind words, he said, were feigned, adding constantly that they did not express the truth, and that he would not answer them. He wrote once to his relations, to ask for some shirts and an outer garment, beseeching them not to abandon him so far as to deny him what was absolutely necessary.

I endeavoured several times to combat his mode of viewing things, and to make him feel that his ideas were mistaken, that his relations had not abandoned him, and, above all, that it could not be supposed that cruelty could enter into any of God's dealings. He always listened to me with incredulity, placing no faith in my words, and appearing to distrust me, and to think that I was conniving with those who wished to deceive him. A lunatic in his division, who was able to give good advice on subjects of piety and morality, once said to me—"It is useless to give him good advice; he will not listen to it; he only attends to the dictates of his own imagination." I have exasperated him, and have seen a natural indignation depict itself on his countenance each time that, feigning for a moment to distrust him, I have appeared to doubt what he related, and to give some degree of credence to the infamous accusation against him. He has often said to me—"Nobody will believe me again. I am an object of horror to every one. What can I do, since God has decreed that all my protestations should be without effect? No one will ever make me admit what is not true, and confess that which has never entered my mind; neither the galleys, torture, nor the scaffold shall ever force me to speak anything but the truth." He often pressed me to send him back to prison, where he should be worse treated, he knew, but where he should be nearer to his executioners, who had devoted him to infamy and execration. Why was he not condemned at first? It was, said he, that he might be made to endure torture. The next time he should be tried he determined not to reply to any question, considering it useless to attempt to justify himself. At other times, in moments of exasperation, he would say, on the contrary, that he would explain everything with the greatest frankness, that he would tell his judges what he thought of their cruelty, that he would even rush upon his advocate, and shut his mouth if he appeared to have any intention of defending him.

On the other hand, I have often seen him more composed in his ideas, and more disposed to listen to my advice—not, indeed, completely freed from his delusions, but apparently staggered in his conceptions, disposed to return to his religious belief, and regretting more than ever the loss of so much happiness. He said then that he had slept well, that his head gave him less pain, and that he felt greatly relieved. These favourable days increased considerably during the second month of his sojourn. I noticed, also, that his physical health improved; he became much stouter, and acquired an appearance of health that left nothing to be desired. At the same time, his mental improvement was not durable; he always returned very rapidly to his habitual restlessness.

*Conclusions.*

[After a full and careful examination, reported at length, of the preceding facts, Dr. Aubanal came to the following conclusions:—]

1st. The accused Louis was predisposed to insanity from birth; and also, as the consequence, of convulsive brain diseases which he suffered from as well in early life as in more recent time.

2nd. The peculiarities of his character always evinced the existence of this natural predisposition.

[The existence of a predisposition in the accused is proved by the mental peculiarities observed during his infancy, and, above all, during the period of his youth passed at the seminary. There, his piety was undoubted, and his general conduct excellent; but there was also remarked in him excessive feebleness of character, infantine manner, numerous oddities and changeableness of mind. The greatest scruples sprung up in his mind as to his religious duties; his imagination was easily excited first by one thing and then by another, without any fixed ideas; he showed an extravagant and even feminine sensibility, was easily impressed, sought to be loved and caressed as a child, and during the hours of recreation was in the habit of committing eccentricities which were commonly remarked by his comrades. . . . . These peculiarities, it is true, do not constitute mental disease. The accused was not yet insane, and no one would have considered him at this time as irresponsible for his actions, although exhibiting very little reflection and some want of judgment. Peculiarities of this kind, however, deserve some attention at the present time. Daily observation teaches us that such facts as these are generally the first moral and intellectual manifestations of folly in those who subsequently become insane. It is rare that one is unable to perceive in cases of congenital or early-acquired predisposition, originalities and eccentricities of various kinds, ardent imagination, weakness of character, imperfect judgment, and other phenomena calling forth general recognition of some serious imperfections in the persons so organised. They have *incomplete organisations*, as it is commonly said, and as one of the witnesses said in speaking of the accused. Individuals so characterised may continue in the same state for life, pursuing their business, living in society and even shining in the world by reason of the pre-eminence of certain faculties; but they are not the less imperfect beings who succumb to the first shock, and present but a feeble resistance to any morbid affection, whether moral or physical. . . . . Such was the condition of the accused from his infancy up to the time when he was invested with the gown. He cannot be considered as insane, since his Superiors had finally decided that he might devote himself to the ecclesiastical state; but he was incontestably predisposed to insanity.]

3rd. The erysipelas, accompanied by delirium, from which he suffered in March 1857, was the determining cause of the mental derangement which befel him; the cephalalgia which followed was indicative of the morbid action which was then commencing.

4th. The malady announced itself, in the first place, by the exaggeration of his singularities, by changes in his habits, and by certain manifestations more or less unreasonable.

5th. It was next characterised by several dominant ideas; but an excessive desire for the extraordinary, a propensity to homicide, and an exaggerated affection for a young comrade were the most decided.

[One can understand up to a certain point the sympathy which may be developed between comrade and comrade, and the intimacy which may result from such an attachment; but a love so ardent and violent as that of the accused, and so characterised by himself—a love which absorbed all his thoughts, which prompted him to write such warmly affectionate letters, once even with the blood from a prick of his finger made voluntarily by himself—a love which continues to the present time with the same intensity, and which he manifests still in the most ardent forms of expression, can only be explained as between man and man in two modes—either as the result of immorality or of a diseased mind.

An immoral desire of this nature, exhibiting itself, so to speak, without reserve before the eyes even of people in the house, is rarely seen but in the *bagnes* or prisons, or in persons of extraordinary perversity. It is then a brutal passion, manifesting itself without refinement or elevation of thought. This was not the case of the accused. He spoke to other pupils of his passion; he spoke of it in terms at once warm and exalted; he confided it even to his Superior. If he had been actuated by any bad thought, would he not have shown more reserve; and, above all, would he have taken a comrade as a confidant and go-between in his correspondence? I cannot believe in the young man's immorality; it is supported by no single fact; his own comrades, even he who so narrowly escaped death at his hands, testify in his favour on this point. And is it to be supposed that the Superior, who was aware of his lively sympathy, would not have sent him away if he had attributed to his passion for a moment such an infamous end?]

6th. His homicidal propensity became at last so strong, that after long resistance he was on the point of yielding to it; it was then that he was tempted to suicide, and that he armed himself with a sword to put his idea of murder into execution.

7th. His passion for the extraordinary, in combination with his homicidal propensity, prompted him to compass the death of his best friend. Other causes—as, for example, the note, contributed to fix the general idea of homicide upon Charles as its object, but they were not the sole or even fundamental causes of the attempted murder.

8th. These dominant ideas influencing the accused, amounted to a real monomania, which, regard being had to the nature of the different series of ideas, might be classed under several denominations, but which I should call homicidal, because this propensity seems to have been the most predominant, and because he ultimately obeyed it in the attempt to murder.

9th. This monomania, followed at intervals by other manifestations more or less indicative of a disordered mind, was not so isolated as its name imports. There were at times, in addition, unmistakable signs of a certain maniacal delirium announcing a general affection of all his faculties.

10th. This mental affection was characterised by numerous remissions. These remissions, which were well marked in the early stage of the disease, naturally tended to disappear as its development progressed.

11th. The disease really existed, although with intermissions or remissions, during the two or three months which preceded the murder. The accused was then perfectly conscious as to the actions of his ordinary life; he was even still conscious as to the nature of the actions to which his dominant ideas were leading him; but his will grew weaker day by day under the domination of these ideas, and he had already committed several acts which show the loss of that faculty, and the existence of great cerebral disturbance.

12th. He was certainly able to appreciate, up to a certain point, the consequences of his actions; he knew perfectly well, for example, that in plunging the weapon into his friend he was about to kill him; but does not the absence of all criminal motive announce that in so doing he fatally obeyed the domination of a delirious idea?

13th. His free-will was profoundly affected, not only by the mastery his foolish conceptions had obtained over his mind, but also as the result of a more general derangement of his faculties.

14th. The circumstances under which the murder was committed are not sufficient to account for it, if viewed as a criminal act. There really does not exist any other fundamental motive for it than that of his monomania.

15th. The accused was not of sane mind for a long time before the murder; he was insane on the day and at the moment of perpetrating it, not forgetting that he premeditated it for ten or twelve hours before.

16th. In prison, and in the asylum, the disease evidenced, with remissions, the same characteristics, but the dominant ideas were not so strong. Some abatement resulted from treatment.

17th. An amelioration may be hoped for under a systematic and prolonged treatment of the disease, but it is doubtful whether it can be radically cured; and if cured, it is very doubtful whether a relapse must not sooner or later occur.

18th. The suggestion that the insanity of the accused is only simulated cannot be sustained; he has never simulated this malady, nor does he simulate at the present moment.

---

